# Net land-atmosphere flows of biogenic carbon related to bioenergy: towards an understanding of systemic feedbacks

**DOI:** 10.1111/gcbb.12071

**Published:** 2013-04-09

**Authors:** Helmut Haberl

**Affiliations:** Institute of Social Ecology Vienna (SEC), Alpen-Adria Universitaet (AAU)A-1070, Schottenfeldgasse 29, Vienna, Austria

**Keywords:** bioenergy, carbon balance, greenhouse gas emissions, long-term socioecological research, material and energy flow analysis, stock-flow dynamics

## Abstract

The notion that biomass combustion is carbon neutral vis-a-vis the atmosphere because carbon released during biomass combustion is absorbed during plant regrowth is inherent in the greenhouse gas accounting rules in many regulations and conventions. But this ‘carbon neutrality’ assumption of bioenergy is an oversimplification that can result in major flaws in emission accounting; it may even result in policies that increase, instead of reduce, overall greenhouse gas emissions. This commentary discusses the systemic feedbacks and ecosystem succession/land-use history issues ignored by the carbon neutrality assumption. Based on recent literature, three cases are elaborated which show that the C balance of bioenergy may range from highly beneficial to strongly detrimental, depending on the plants grown, the land used (including its land-use history) as well as the fossil energy replaced. The article concludes by proposing the concept of GHG cost curves of bioenergy as a means for optimizing the climate benefits of bioenergy policies.

## Introduction

Per unit of energy, combustion of biomass releases approximately as much carbon dioxide (CO_2_) to the atmosphere as combustion of coal. Depending on their respective qualities, biomass and coal both emit ∼0.1 g CO_2_ per Joule (±15%). CO_2_ emissions per unit of energy of oil products and natural gas are substantially lower (∼−25% and ∼−50% respectively). Many laws and regulations (e.g., the European Emission Trading System or the European Renewable Energy Directive) nevertheless build on accounting systems that ignore these CO_2_ emissions, thereby explicitly or implicitly treating biomass combustion as if it were carbon neutral vis-à-vis the atmosphere (Searchinger *et al*., 2009; Searchinger, 2010; Haberl *et al*., 2012). This is commonly justified by arguing that biomass combustion only returns CO_2_ to the atmosphere that had previously been sequestered during plant growth.

However, this ‘carbon neutrality’ assumption is an oversimplification that can result in incorrect GHG accounts (Fargione *et al*., 2008; Bird *et al*., 2012; Holtsmark, 2012). A proposed solution is to account for the CO_2_ resulting from biomass combustion and subtract a credit for the CO_2_ removed from the atmosphere as a result of bioenergy production (Searchinger *et al*., 2009; Bird *et al*., 2012; Haberl *et al*., 2012). The question is how this credit can be calculated, given the multitude of systemic feedbacks in land systems related to biomass production. This commentary aims to contribute to this question by re-phrasing the problem based on ecosystem theory, above all ecosystem energetics and succession theory, as well as socioecological mass-balancing principles. Only land-based bioenergy systems are considered; algae are beyond its scope. ‘Bioenergy’ denotes solid, liquid or gaseous combustible materials derived from biomass used to produce technical energy (excluding food and feed). The article only discusses carbon (C) flows originating in plant growth, i.e. consumption of plants and plant parts in heterotrophic food webs (including humans and livestock) as well as combustion. Other greenhouse gas (GHG) emissions such as those resulting from fossil fuels used in agriculture or biomass conversion and other gases (N_2_O or CH_4_) are outside its scope.

## Conceptual considerations

### Carbon flows in ecosystems

**Fig. 1:**
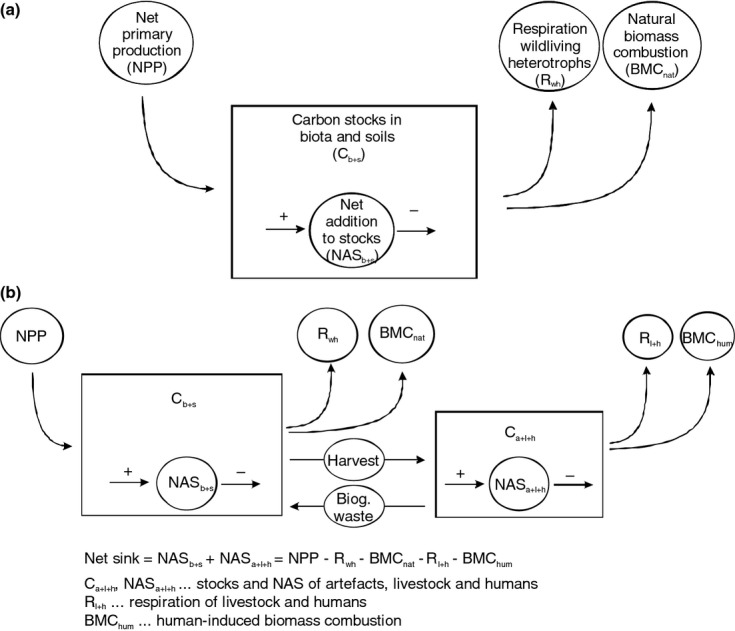
Stylized representation of stocks and flows of C in land ecosystems (a) without humans, (b) with humans. The squares denote stocks (kg C) and the circles flows (kg C year^−1^). Complexities arising from plant metabolism are excluded from the graph by starting with net primary production (NPP) which is defined as gross C absorption by plants (GPP) minus C released by plant respiration.

Plants absorb inorganic C in the form of CO_2_ from the atmosphere and convert it to organic C, i.e. C incorporated in myriads of chemical compounds in living tissues. This process is based on the conversion of sunlight into chemically stored energy in photosynthesis. The total amount of C fixed by plants is denoted as Gross Primary Production or GPP. The plants use parts of the assimilated C for their metabolism (plant respiration); GPP minus plant respiration is Net Primary Production (NPP). NPP is organic C available for (1) consumption by heterotrophs (i.e. all organisms not capable of photosynthesis – animals, microorganisms and fungi), (2) natural wildfires and (3) build-up of C stocks in biota and soils, i.e. C stored in living (or recently living) organisms as well as organic C in the soil (soil organic matter, SOM). These principal C stocks and flows are summarized in Fig. 1a.

It is useful to denote as ‘sink’ a flow from the atmosphere to the ecosystem and as ‘source’ a flow in the opposite direction (‘directed’ terms are used throughout as they facilitate discussion). The carbon sink of biota and soils is equal to the net addition to stocks of biota and soils (NAS_b+s_) which can be written as


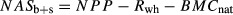


where R_wh_ is the respiration of wild-living heterotrophs and BMC_nat_ are natural fires. However, both R_wh_ and BMC_nat_ are difficult to measure. Hence, most estimates of the terrestrial carbon sink are based on accounts of the carbon stocks of biota and soils (C_b+s_) at different points in time, i.e. on a direct quantification of NAS_b+s_. NAS_b+s_ may be negative, in which case the ecosystem is a C source.

Because the atmospheric C concentration was largely stable over long periods in the earth's history, most researchers tend to believe that C_b+s_ is more or less constant (i.e. NAS_b+s_ close to zero) over larger regions and longer time periods. This is not the case in smaller spatial scales (see below), for short periods of time, for some ecosystems (e.g. peat bogs) as well as during periods of climatic perturbations or other natural changes in the earth system (Luyssaert *et al*., 2008; Houghton *et al*., 2012). If C stocks are constant, NPP equals the sum of heterotrophic respiration and natural fires.

### Socioecological systems

Stocks and flows in a socioecological system are shown in Fig. 1b. Humans harvest biomass for food and livestock feed production, raw materials and energy. Harvested biomass is used to build-up socioeconomic C stocks (Lauk *et al*., 2012), i.e. C in human bodies and livestock as well as biogenic C in artefacts (e.g. timber in buildings, paper in books). Outflows of the socioeconomic compartment are respiration of humans and livestock (R_l+h_), i.e. the C from food and feed metabolized by humans and livestock, as well as biomass combustion, i.e. bioenergy and other human-induced fires (BMC_hum_). Organic materials (e.g. organic fertilizers or wastes) flow back from society to the ecosystem where they decompose more or less rapidly, thereby releasing C as part of R_wh_. The net C sink equals stock changes, in this case NAS_b+s_ + NAS_a+l+h_, which equals the difference between NPP and respiration plus biomass combustion of natural and human compartments. Whether coupled socioecological systems act as a C source or sink depends on a various natural and socioeconomic factors, many of which involve substantial complexities regarding stock/flow interactions; legacy effects, systemic feedbacks and time lags.

One important aspect to consider is NPP. In natural ecosystems, NPP is determined by natural factors such as climate, soil, solar irradiation and atmospheric C content. This may still be largely the case for landscapes inhabited by hunter–gatherer societies, but at least since the Neolithic revolution, i.e. the advent of agriculture, NPP is co-determined by natural and socioeconomic factors, e.g. land management in agriculture and forestry. NPP of human-used lands (denoted as NPP_act_ for NPP of actual, i.e. currently prevailing vegetation) usually differs from that of natural terrestrial ecosystems (denoted as NPP_pot_, i.e. the NPP of potential natural vegetation assumed to exist in the absence of humans). Land use may reduce or increase NPP. In most pre-industrial land systems, NPP_act_ is smaller than NPP_pot_. Even in intensive agriculture, NPP_act_ is sometimes lower than NPP_pot_, although in some regions, e.g. in north-west Europe, the NPP_act_ of agro-ecosystems surpasses NPP_pot_ on regional scales (Haberl *et al*., 2007).

Figure 1b shows the complexity involved in estimating the net effect of biomass combustion on atmospheric CO_2_: the net C balance of biomass combustion cannot be assessed in isolation – this is only possible when considering all other processes, in particular food and fibre production. The ‘carbon neutrality’ assumption is only valid if the growth of plants that are used to produce bioenergy is entirely independent of all other flows or if the sum of all feedbacks is zero, and if growing and burning the plants is not related with any C stock changes in ecological and socioeconomic systems. This may be the case in a static situation with little changes in food, feed and bioenergy production, but it is unlikely if bioenergy (or food and feed) production is strongly increased. Evaluating such dynamic cases is more complex because it is necessary to take changes in C stocks and flows into account.

## A long-term socioecological (LTSER) perspective

**Fig. 2:**
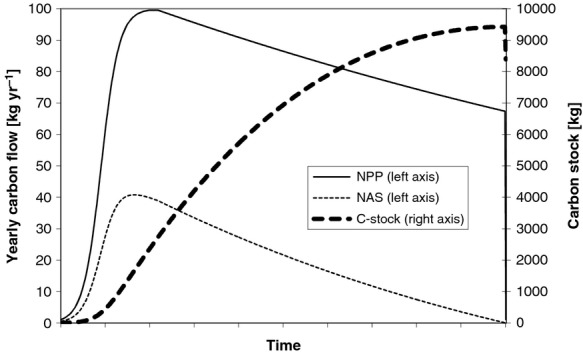
Stylized representation of the development of stocks and flows of carbon in an ecosystem at the plot scale over time. NPP … net primary production, NAS … net addition to stocks, i.e. carbon sequestration of biota and soils, C stock … carbon stock in biota and soils. Source: own graph (after Odum, 1969).

**Fig. 3:**
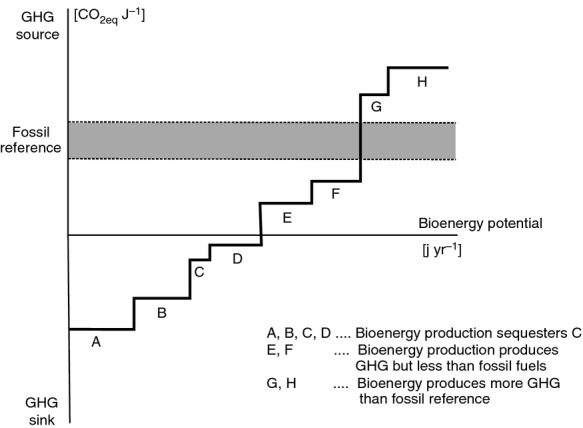
Conceptual diagram of a greenhouse gas cost curve for bioenergy. The curve describes the marginal costs of increasing bioenergy production and use. It can be constructed by evaluating the full GHG effects per unit of energy of options A, B, C, etc., to increase bioenergy production and use. In addition to the land–use-related C balance issue, such a curve would also need to consider GHG flows that were beyond the scope of this commentary, i.e. C emissions from fossil energy use and non-CO_2_ GHG emissions.

According to ecological succession theory (Odum, 1969), stocks and flows of C in ecosystems follow a distinct time path: If plants colonize bare soil, a succession starts which is characterized by changes in stocks and flows of C (Fig. 2). NPP is thought to increase according to a logistic function, i.e. it starts off with exponential growth that slows down in approaching a maximum, gradually declining afterwards. Heterotroph respiration (R_wh_) and natural fires (BMC_nat_) consume a part of the NPP, therefore carbon sequestration (i.e. net addition to stocks in biota and soils, NAS_b+c_) is smaller than NPP. Over time, C outflows approach NPP and NAS converges to zero. The carbon stock (C_b+s_) depicted on the secondary axis in Fig. 3 grows at an increasing rate in the beginning, but later growth slows down and the C stock eventually stabilizes. Towards the end, the ecosystem is often dominated by trees. When trees die (due to age or during natural fires), a considerable proportion of the C stock is released in short time (‘slow in, fast out’; Körner, 2003) and succession starts again. Figure 3 summarizes C stocks and flows during a succession at the plot scale during succession.

At a large scale and over longer time periods, one would expect to find a mosaic of ecosystems in different succession stages in which the slow influx of C of earlier succession stages is more or less balanced by the fast C outflow following breakdown of trees, i.e. C stocks are constant and NPP is balanced by heterotrophic respiration and natural fires on larger scales. This pattern probably holds for most natural landscapes, although there are exceptions such as peat bogs that accumulate C perhaps even on geological time scales (Körner, 2003).

The relations between C stocks and flows, as well as the mass of C stocks in different pools (above- and belowground vegetation, soil) differ substantially between ecosystem types. Use of the land for human purposes usually entails a replacement of the original vegetation cover with other species. The most prominent example is replacement of forests with cropland and grazing areas, which reduces C stored in biota and soils (C_b+s_) (Houghton *et al*., 2012). In the case of Austria, the C stock of the actual vegetation is ≈40% smaller than that of the original vegetation (Erb *et al*., 2008). Total (above- and belowground) C stocks of cropland in Austria are estimated at 7.3 kg C m^−2^ which is only one-third of the C stock of commercial forests (21.2 kg C m^−2^) and even smaller if compared with non-commercial forests (27 kg C m^−2^) which may be similar to potential natural vegetation (Gingrich *et al*., 2007). Over the period 1830–2000, abandonment of cropland and grazing lands combined with regrowth of forests resulted in considerable C sequestration: C stocks grew by ≈20% in that period (Erb *et al*., 2008). That is, even if land use is C neutral in terms of yearly flows, it is often associated with past C losses, i.e. a ‘C debt’ (Fargione *et al*., 2008). In many cases, the C loss is reversible, i.e. if land is abandoned, C stocks grow (Erb *et al*., 2008). Continuation of land use may hence incur ‘opportunity costs’ in terms of the foregone C sequestration potential of the land.

Taking these interdependencies into account, Fig. 1b can be used to discuss the system-level effects that need to be evaluated when analyzing the effects of changes in bioenergy use: Emissions resulting from altered bioenergy production and use (ΔBMC_hum_) are compensated by plant growth if and only if


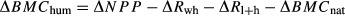


where all changes (denoted by Δ) in the C flows on the right-hand side of the equation are related to the production of the bioenergy (i.e. are ‘additional C’ in the sense of Searchinger, 2010): Biomass combustion is C neutral only if biomass burned for energy provision results from increased plant growth (ΔNPP) or from reduced respiration of wild-living animals, livestock or humans, or from reduced natural biomass combustion so that all these changes in flows fulfil the above-cited equation.

This may be the case, but in many instances emissions from biomass combustion will be higher or lower than the term on the right-hand side of the equation. If they are lower, bioenergy has ‘negative’ C emissions, i.e. helps to reduce C flows to the atmosphere even if no fossil-fuel emissions are replaced. If they are higher, biomass combustion adds C to the atmosphere. The C emissions per unit energy may be smaller or larger than those of fossil energy-derived fuels. If they are larger, then substitution of biomass for fossil fuels increases C flows into the atmosphere, if they are smaller, C flows are reduced, but by less than 100%. (GHG emissions of biomass production – agriculture, machinery, conversion processes – as well as those associated to producing fossil energy-derived fuels are beyond the scope of this article, but need to be considered to allow comprehensive comparisons. If C emissions from combustion are captured with carbon capture and storage [CCS], this also changes the picture; CCS is also beyond the scope of this paper.)

It is difficult to directly evaluate whether C flows resulting from biomass combustion are balanced by other flows due to the complexity of the land-use/biomass flow system. One can often only evaluate the C balance of the whole land-use/biomass flow system. Moreover, the ‘C debt’ issues raised above suggest that a general ‘C neutrality’ assumption would be inappropriate even if the C balance is close to zero: continuation of land use often incurs an ‘opportunity C cost’ compared with reforestation which would be neglected by inferring C neutrality from constant stocks of the current system.

While the direct quantification of the C balance of present bioenergy systems seems difficult, one can use Fig. 1b for two other purposes: (1) one can estimate marginal C costs of increasing bioenergy production and use, and (2) one can estimate C costs of present bioenergy use by calculating the opportunity costs its production incurs. In both cases, systemic feedbacks – e.g., changes in land-use competition between food and energy production (Lambin & Meyfroidt, 2011) – likely and strongly affect the outcome (Haberl *et al*., 2011; Smith *et al*., 2013).

## Possible orders of magnitude – some examples

### Bioenergy production on degraded land

Long-term experiments on degraded and abandoned nitrogen-poor land in the US suggest considerable potentials to produce carbon-negative bioenergy (Tilman *et al*., 2006). The experiment was conducted in Cedar Creek, Minnesota, 1994–2005. Plots were managed at low intensity, irrigation and fertilization were only provided once when plots were established. Sowing the plots with 16 species resulted in highest biomass yields, numbers cited here refer to that treatment. Biomass yield was 0.19 kg C m^−2^ year^−1^ which equals 6.8 MJ m^−2^ year^−1^ primary energy and 2.8 MJ m^−2^ year^−1^ final energy (synfuel and electricity) in the most efficient conversion pathway analysed. In addition to delivering energy, the land also sequestered 0.27 kg C m^−2^ year^−1^; the authors estimated that C sequestration might last several decades. The land was abandoned and it may hence be assumed that there are little, if any, feedbacks, and because it was severely degraded, opportunity C costs are probably small or absent. Studies on degraded, salinized Australian drylands also suggest that considerable amounts of biomass (up to 0.23 kg C m^−2^ year^−1^) could be produced by high-density Eucalyptus plantations which would contribute to restoring degraded land, i.e. improve food production, while simultaneously sequestering C (Harper *et al*., 2012; Sochacki *et al*., 2012). Of course these studies are site-specific and should not be extrapolated to larger regions without further analysis of the availability and suitability of the land for such management schemes.

### Opportunity C costs of bioenergy from cropland

Reforestation of European croplands sequesters ≈0.32 kg C m^−2^ year^−1^ (Smith *et al*., 2000). Short-rotation coppice (SRC) can yield a GHG reduction of almost 0.5 kg C_eq_ m^−2^ year^−1^ if it replaces coal (Smith *et al*., 2013). If other energy carriers are replaced, benefits are smaller. Moreover, empirical studies questioned the assumption that increased bioenergy production results in a 1:1 reduction in fossil fuels, e.g. due to rebound effects (York, 2012). Such effects would reduce the benefit of SRC. However, C sequestration saturates after decades or centuries (see Fig. 2), while SRC production may continue, hence long-term benefits would be larger for SRC. If bioenergy or afforestation compete with food production for land this may result in indirect land-use changes with considerable effects on the C balance (Searchinger, 2010; Chum *et al*., 2012). Land-use competition may affect food prices and motivate switches to less land-demanding diets which could have climate benefits. This effect would be inacceptable in poor regions where it would contribute to hunger and malnutrition (Searchinger, 2010). Even without full evaluation of feedbacks it seems unlikely that ethanol from maize (or other similar liquid biofuels) would stand the ‘opportunity cost’ test. According to Chum *et al*. (2012), substituting ethanol from maize for gasoline reduces emissions by 45% with an average ethanol yield of 7.5 MJ m^−2^ year^−1^, which translates into a GHG reduction potential of 0.074 kg C_eq_ m^−2^ year^−1^, i.e. less than a quarter of the C sequestration potential of that area.

### C costs of additional harvest in forests

In many forests worldwide harvest is smaller than increment, suggesting that increased harvests would be sustainable. But this does not imply that increased harvest would be C neutral. Recent modelling work (Hudiburg *et al*., 2011; Holtsmark, 2012) suggests that permanently increasing harvest levels in forests results in a permanent reduction of the equilibrium C stock of the forest, i.e. in a C debt. That is, increased harvest reduces C sequestration in the growing forest compared to a scenario with lower harvest levels. Depending on the forest management regime as well as on the assumptions regarding the fossil energy carrier that is replaced, this C debt may require decades or centuries to be repaid even if the harvest level is sustainable. Calculations by Holtsmark (2012) suggest that a permanent increase of wood harvest in Norwegian forests by 30% compared with the current harvest level reduces the C stock by ≈1 kg C m^−2^ after 100 years and by ≈ 1.3 kg C m^−2^ after 200 years. If one assumes that the additional wood harvested is used to produce pellets that replace coal in a power plant, the C reduction per unit area (of the whole forest) is ≈0.0067 kg C m^−2^ year^−1^; the C debt is repaid after ≈200 years (it takes much longer if the wood is used to produce liquid fuel). Measures to increase the productivity of the forest (faster growing trees) can reduce the payback period by about one-third. (These calculations consider energy inputs required in biomass processing; in forests these energy inputs are small compared with the energy in the wood; see the supplement to Holtsmark, 2012.)

## Discussion and conclusions

Conceptual considerations in sections 2–3 as well as the examples in section 4 show the deficiencies of the ‘carbon neutrality’ assumption, i.e. the assumption that C released when biomass is burned is compensated by plant growth and hence need not be counted when accounting for the GHG emissions of bioenergy. Nevertheless, this assumption is still explicitly or tacitly taken for granted in much research, policy-making and even laws (Searchinger, 2010; Bird *et al*., 2012; Haberl *et al*., 2012). Correcting the errors introduced by that assumption is a key prerequisite for prioritizing bioenergy options that are either C negative (A–D in Fig. 3) or at least beneficial compared with fossil fuels (E, F), and avoiding detrimental ones (G, H).

Constructing GHG cost curves of bioenergy as conceptualized in Fig. 3 is a complex endeavour due to the manifold feedback processes in the land system. As shown in this article, GHG cost curves of bioenergy depend on (a) land-use history and (b) future changes in the entire land-use system, above all food and feed production. GHG cost curves of bioenergy are contingent on the assumptions on diets, food crop yields (including climate-change feedbacks on yields), livestock feeding efficiencies, land requirements of biodiversity conservation, fibre demand and many other factors (e.g. Haberl *et al*., 2011). A particularly important feedback to be considered is that other ecosystem services beyond C may be affected by bioenergy policies (Smith *et al*., 2013). More research into these systemic feedback processes should hence have a high priority for underpinning sustainable bioenergy policies that maximize climate benefits of bioenergy.
